# Effect of meteorological variables on the incidence of hand, foot, and mouth disease in children: a time-series analysis in Guangzhou, China

**DOI:** 10.1186/1471-2334-13-134

**Published:** 2013-03-13

**Authors:** Yong Huang, Te Deng, Shicheng Yu, Jing Gu, Cunrui Huang, Gexin Xiao, Yuantao Hao

**Affiliations:** 1 Department of Medical Statistics and Epidemiology & Health Information Research Center & Guangdong Key Laboratory of Medicine, School of Public Health, Sun Yat-sen University, Zhongshan Road II, Guangzhou, Guangdong Province, 510080, China; 2 Chinese Center for Disease Control and Prevention, Beijing, 102206, China; 3 School of Public Health & Institute of Health and Biomedical Innovation, Queensland University of Technology, Brisbane, QLD, 4059, Australia

**Keywords:** Children, Generalized additive model, Hand, foot, and mouth disease, Meteorological variables, Time series analysis, China

## Abstract

**Background:**

Over the last decade, major outbreaks of hand, foot, and mouth disease (HFMD) have been reported in Asian countries, resulting in thousands of deaths among children. However, less is known regarding the effect of meteorological variables on the incidence of HFMD in children. This study aims at quantifying the relationship between meteorological variables and the incidence of HFMD among children in Guangzhou, China.

**Methods:**

The association between weekly HFMD cases in children aged <15 years and meteorological variables in Guangzhou from 2008 to 2011 were analyzed using the generalized additive model (GAM) and time-series method, after controlling for long-term trend and seasonality, holiday effects, influenza period and delayed effects.

**Results:**

Temperature and relative humidity with one week lag were significantly associated with HFMD infection among children. We found that a 1°C increase in temperature led to an increase of 1.86% (95% CI: 0.92, 2.81%) in the weekly number of cases in the 0–14 years age group. A one percent increase in relative humidity may lead to an increase of 1.42% (95% CI: 0.97, 1.87%) in the weekly number of cases in the 0–14 years age group.

**Conclusions:**

This study provides quantitative evidence that the incidence of HFMD in children was associated with high average temperature and high relative humidity. The one-week delay in the effects of temperature and relative humidity on HFMD is consistent with the enterovirus incubation period and the potential time lag between onset of children’s sickness and parental awareness and response.

## Background

Hand, foot, and mouth disease (HFMD) is an infectious gastrointestinal disease commonly caused by enterovirus 71 (EV71) and coxsackievirus A16 (Cox A16) and occurring mainly in children under 5 years old [1,2]. HFMD is primarily transmitted via fecal-oral route, respiratory droplets, contact with blister fluid of infected individuals or general close contact with infected individuals [2]. In recent decades, Asian countries have experienced frequent and widespread HFMD outbreaks with deaths predominantly among children. However, currently there is no vaccine or effective curative treatment available. The frequency of HFMD outbreak is also expected to increase in future due to continued viral mutation, climate change, and the lack of health resources and effective surveillance systems in some countries [2-5].

Seasonality in the incidence of HFMD has been seen in a number of countries. In Japan, a seasonal peak was detected during the summer months [6,7], while in Finland most HFMD cases were reported in autumn [8]. In several Asian countries (Singapore, Malaysia, Hong Kong, Taiwan and mainland China), epidemics usually peak in the late spring/early summer, along with a second small peak in late autumn/early winter [5,9-12]. The seasonality of HFMD suggests that meteorological variables might be influential in the spread of the disease.

The relationship between meteorological variables and HFMD has been documented in a few studies and the findings are inconsistent [6,13-15]. For example, a Singapore study showed that weekly maximum temperature above 32°C elevated HFMD incidence and a Hong Kong study also supported the positive association [13,14]; whereas another study in Japan found that the number of days per week of the average temperature above 25°C was negative associated with HFMD incidence [15]. In addition, a recent study in Japan found non-significant association between rainfall and HFMD, contrary to findings in Singapore [6,13]. Moreover, high wind speed was shown to be a risk factor for HFMD in the Hong Kong study [14], but no other study supports these findings.

In China, a serious outbreak of HFMD occurred among infants and young children, causing 6,049 cases and 22 deaths in Fuyang City, Anhui Province between March 1 and May 9, 2008 [16]. Since May 2008, the Chinese Ministry of Health has categorized HFMD as a Class “C” infectious disease, and started monitoring the disease via the National Communicable Disease Surveillance Network. Unlike other sentinel surveillance data, the data collected from this network is population-wide, which can be more representative for the actual HFMD epidemic. A recent data quality inspection report has demonstrated that the data are of decent quality, especially in the eastern regions of China, with reporting completeness of 99.84% and accuracy of the information reported of 92.76% [17]. In the past four years, the research on this data of HFMD focused on descriptive epidemiology, seroepidemiology, virology, pathogenesis and treatment aspects [18-22]. However, there is a need for more frequent statistical analysis of the relationship between meteorological variables and HFMD incidence in China. Furthermore, it is not clear how meteorological variables influence HFMD among children of different ages. There is an urgent need to investigate such relationships which can help prediction of future outbreak and evaluation of mitigation strategies.

We carried out a time-series analysis of the association of weekly HFMD cases with meteorological variables in Guangzhou, China using data from Chinese National Communicable Disease Surveillance Network. The aim of this study was to quantify the relationships between meteorological variables and HFMD incidence among children.

## Methods

### Ethics statement

No work with human subjects was directly involved in our research. The HFMD case data were extracted from Chinese Center for Disease Control and Prevention (China CDC) weekly reports, which are governmental reports summarizing counts of patients diagnosed at health care facilities with a variety of disease. All individual-level data were anonymous. Permission to conduct the research was granted by IRB at China CDC and Sun Yat-sen University.

### Study site

Guangzhou is the capital city of Guangdong Province and the third biggest city in China, about 200 km from Hong Kong. It has a population density of 1,710 persons per km^2^ (in 2010: population = 12,709,600 persons; land size = 7434.4 km^2^). Guangzhou features a subtropical monsoon climate, with an annual average temperature of 22°C and relative humidity of 75% [23].

### Surveillance data of hand, foot, and mouth disease

Weekly reported cases of HFMD in Guangzhou from May 2008 to December 2011 were obtained from the National Center for Public Health Surveillance and Information Services, China CDC. The clinical criteria for diagnosis of HFMD cases was provided in a guidebook published by the Chinese Ministry of Health in 2009 [24]. Patients with the following symptoms are defined as having HFMD: fever, papules and herpetic lesions on the hands or feet, rashes on the buttocks or knees, inflammatory flushing around the rashes and little fluid in the blisters, or sparse herpetic lesions on oral mucosa. Clinical diagnoses of HFMD are strictly examined and verified by various levels of CDC. At the provincial level, at least 10 cases were serotyped per month. Serotyping and sequencing were performed at provincial surveillance laboratories with quality control from China CDC.

According to our data, 99.5% HFMD cases were children aged 0–14 years. Therefore, we focused on the incidence of HFMD among children aged 0–14 years in this study. The form of child care impacts HFMD infection. In China, children aged 0–2 years are usually cared for at home, 3–5 years attend kindergartens, and 6–14 years go to school. Children aged <1 year obviously differ from those aged 1–2 years in daily activity. To investigate which age groups are the most susceptible to meteorological changes, we conducted the analyses for different age subgroups (<1, 1–2, 3–5, and 6–14 years) using the models of the best fit to the overall data.

### Meteorological data

We obtained data of weekly meteorological variables from the National Meteorological Information Center (http://cdc.cma.gov.cn/). The data were collected from a meteorological station in Guangzhou city. Four weekly meteorological variables were included in this study: average temperature, average relative humidity, average rainfall and average wind speed.

### Statistical analysis

Generalized additive model (GAM) with a negative binomial family was used to estimate associations between weekly HFMD cases and average temperature, relative humidity, rainfall and wind speed. GAM is useful in identifying exposure-response relationships for many types of data, particularly in exploring nonparametric relationships [25]. The distributional assumption of negative binomial accounts for over-dispersion in count data better than the traditional Poisson assumption [26].

We first developed a basic temporal model for HFMD cases excluding meteorological variables. To adjust for long-term trends and seasonality, we included penalized spline functions of time in the model. The number of degrees of freedom (*df*) for time was determined by minimizing of the sum of the absolute values of partial autocorrelation function (PACF) of residuals for lags up to 30 weeks [27-29]. HFMD activities could be different in school holiday vs. non-school holiday periods [30], so holiday (in summer and winter) effects were controlled in all models as indicator variables. During the H1N1 influenza epidemic in 2009, the transmission of respiratory viruses among children in Guangzhou was greatly reduced by massive use of face masks, school closures and reduction of outdoor activities. Due to different epidemic levels of influenza, the HFMD epidemic can be influenced between the different years because media attention may lead to behavior changes. Therefore, we incorporated indicator variables for years to account for between-year changes in different epidemic levels of influenza.

Second, we built meteorological models based on the temporal models. If the incidence of HFMD was indeed influenced by the changes in meteorological variables, the delayed effects of meteorological variables on HFMD should be considered, particularly taking into account the incubation period of HFMD. Assuming that the incubation period for HFMD was about 3 to 7 days, we examined the effect of meteorological variables with different lag time including both single-week lag (from Lag0 to Lag2) and multi-week lag (Lag0–2) to capture immediate effects and cumulative effects, respectively. For example, Lag0 and Lag1 correspond to the current-week and previous-week meteorological variables while Lag0–2 refers to 3-week average of meteorological variables in the current and previous two weeks. Penalized splines with 3 degrees of freedom were used to fit the association between case incidence and each of the meteorological variables.

To control for the autocorrelation, the model’s residuals were examined for serial correlation using PACF. If significant serial correlation remained on certain lags of the PACF, we incorporated an autoregressive term of that order into the model.

The results were reported as percentage changes in the weekly number of HFMD cases per unit increase in meteorological variables, and associated 95% confidence intervals (95% CI). All statistical analyses were performed using R 2.15.0 [31].

## Results

### Descriptive statistical results

A total of 100,875 children aged 0–14 years (14,258 aged <1 years, 52,115 aged 1–2 years, 30,284 aged 3–5 years and 4,218 aged 6–14 years) were infected with HFMD during the study period from May 2008 to December 2011 (Table 1).

**Table 1:** Descriptive statistics for weekly HFMD cases and meteorological variables, 2008–2011

**Variables**	**Total**	**Mean**	**SD**	**Min**	**Median**	**Max**
Cases, 0–14	100,875	528.1	564.4	7.0	329.0	2294.0
Cases, <1	14,258	74.6	83.0	0.0	39.0	350.0
Cases, 1–2	52,115	272.9	282.3	5.0	175.0	1159.0
Cases, 3–5	30,284	158.6	181.9	1.0	89.0	757.0
Cases, 6–14	4,218	22.1	28.9	0.0	11.0	161.0
Temperature (°C)	–	22.9	6.0	8.1	24.9	32.1
Humidity (%)	–	72.3	10.1	37.7	73.6	90.1
Rainfall (mm)	–	5.5	8.2	0.0	2.4	40.3
Wind speed (m/s)	–	1.8	0.7	0.8	1.5	4.4

**Figure 1:**
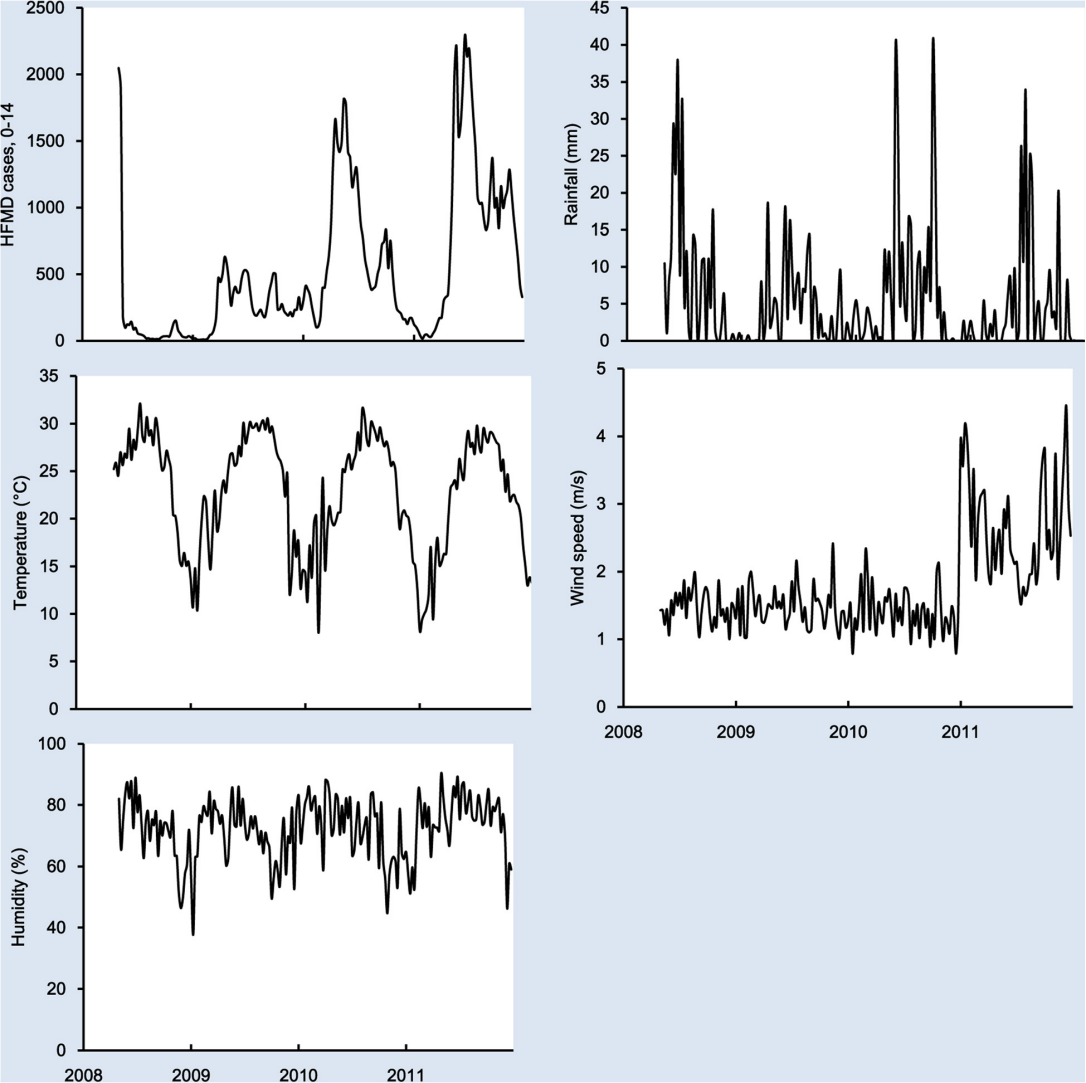
Seasonal distribution of weekly HFMD cases and meteorological variables in Guangzhou, 2008–2011.

Figure 1 shows the seasonal distribution of weekly HFMD cases and meteorological variables in Guangzhou. There was an obvious seasonal pattern and upward trend in the number of HFMD cases for children aged 0–14 years. A summer peak was observed in April–June with a second smaller peak in September and October.

For all age groups, the weekly HFMD cases were positively correlated with temperature, relative humidity, rainfall and wind speed, except that wind speed were not significantly correlated with HFMD cases in the <1 and 6–14 years age groups (Table 2). However, there is a high correlation between relative humidity and rainfall (*r* = 0.65, *P* < 0.001). Thus, relative humidity and rainfall were not included in the same model due to potential collinearity (Table 2).

**Table 2:** Spearman correlation coefficient between weekly HFMD cases and meteorological variables, 2008–2011

**Variables**	**Cases, 0–14**	**Cases, <1**	**Cases, 1–2**	**Cases, 3–5**	**Cases, 6–14**	**Temp (°C)**	**Humidity (%)**	**Rainfall (mm)**
Temperature (°C)	0.28*	0.37*	0.29*	0.19*	0.30*	–		
Humidity (%)	0.27*	0.28*	0.27*	0.26*	0.25*	0.18*	–	
Rainfall (mm)	0.25*	0.28*	0.25*	0.22*	0.26*	0.39*	0.65*	–
Wind speed (m/s)	0.16*	0.13	0.17*	0.16*	0.13	-0.09	0.01	-0.11

* Statistically significant.

### Regression results

Wind speed was initially considered in the model, but no relationship with the HFMD incidence was detected (*P* > 0.1). So, we did not include wind speed in the final GAM models.

**Figure 2:**
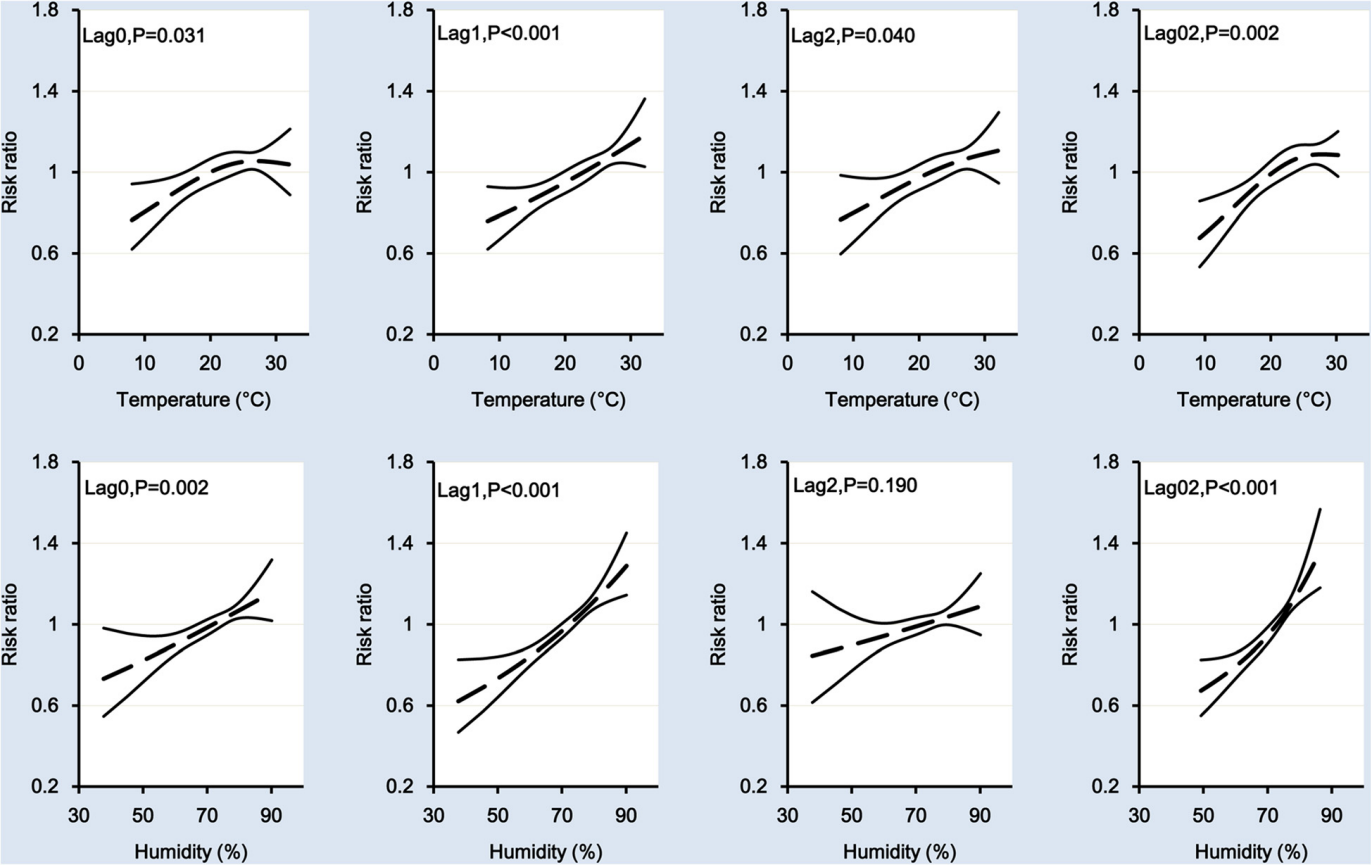
Risk ratios for the association of weekly HFMD cases among children aged 0–14 years by temperature and relative humidity on single-week lags and multi-week lag, respectively.

Risk ratio of weekly HFMD incidence (relative to the risk at the mean value of the covariate) among the 0–14 years age group was plotted against covariate values for both temperature and relative humidity with different lag settings in Figure 2. Among single-week lag models, the effects of both temperature and relative humidity with one week lag on HFMD were statistically significant, with the most dramatic changes observed in risk ratio. We also fitted models with temperature and rainfall, but the results were similar to models with temperature and relative humidity (not shown), and rainfall is therefore not included in our final models.

For Lag0 model and Lag0–2 model, the risk ratio increased sharply before 25°C but became relatively flat afterwards. However, the trends seemed linear for Lag1 model and Lag2 model, which was supported by the chi-square test for linearity. As a result, we used piecewise linear splines for Lag0 and Lag0–2 models, and linear regression for Lag1 and Lag2 models to estimate the effects. In the piecewise linear spline model, we used a breakpoint at 25°C for temperature. Table 3 shows the percentage increases in HFMD cases associated with changes in temperature and relative humidity for single-week lags (from Lag0 to Lag2) and multi-week lag (Lag0–2). Among single-week lag models, the Lag1 models had the best fit with the highest coefficients of determination (Lag0: 0.91, Lag1: 0.94, Lag2: 0.92).

**Table 3:** The effects of temperature and relative humidity on HFMD cases in children aged 0–14 years, 2008–2011

**Variables**	***R***^**2**^	**% increase**	**95% CI**	***P *value**
**Lag0 model**				
Temperature < =25°C	0.91	1.72	(0.35, 3.11)	0.014
Temperature >25°C		-0.43	(-3.71, 2.96)	0.800
Humidity		0.92	(0.46, 1.39)	<0.001
**Lag1 model**				
Temperature	0.94	1.86	(0.92, 2.81)	<0.001
Humidity		1.42	(0.97, 1.87)	<0.001
**Lag2 model**				
Temperature	0.92	1.46	(0.34, 2.58)	0.010
Humidity		0.50	(-0.01, 1.02)	0.052
**Lag0-2 model**				
Temperature < =25°C	0.94	2.69	(1.02, 4.39)	0.002
Temperature >25°C		-0.93	(-4.54, 2.82)	0.623
Humidity		2.06	(1.42, 2.71)	<0.001

The effects of temperature and relative humidity on HFMD incidence were strongest in the Lag1 models, compared to the Lag0 and Lag2 models. The Lag1 model suggested that a potential 1°C increase in temperature was associated with a 1.86% (95% CI: 0.92, 2.81%) increase in HFMD incidence in those aged 0–14 years. A one percent increase in relative humidity led to a 1.42% (95% CI: 0.97, 1.87%) increase in HFMD incidence in those aged 0–14 years. Coefficients of determination of the multi-week lag models were similar to that of the Lag1 models, but the effects of temperature and relative humidity were higher.

We used Lag1 models (linear model) to further estimate the effects of temperature and relative humidity among different age groups. Our analysis revealed that a potential 1°C increase in temperature was associated with 3.1% (95% CI: 1.6, 4.7%), 2.1% (95% CI: 1.1, 3.0%), 1.6% (95% CI: 0.5, 2.8%) and 2.3% (95% CI: 0.5, 4.2%) increase in HFMD incidence in those aged <1 years, 1–2 years, 3–5 years and 6–14 years, respectively. A one percent increase in relative humidity led to 1.7% (95% CI: 1.0, 2.3%), 1.4% (95% CI: 0.9, 1.9%), 1.6% (95% CI: 1.0, 2.1%) and 1.4% (95% CI: 0.5, 2.2%) increase in HFMD incidence in those aged <1 years, 1–2 years, 3–5 years and 6–14 years.

## Discussion

In the last decade, Asian countries have seen frequent and widespread hand, foot, and mouth disease (HFMD) outbreaks among children, but few studies have explored the relationship between meteorological variables and HFMD incidence. To the best of our knowledge, this is the first study in China using population-wide monitoring data to evaluate the effects of meteorological variables on HFMD incidence at different time lags among children of different ages. This study could provide guidance to policy makers, health agencies and local communities in predicting peak time and scale of HFMD outbreaks, improving surveillance, choosing prevention and control strategies, and allocating health resources.

During the four-year study period, HFMD cases in Guangzhou city increased every year. In particular, there were obvious increases in the number of cases in the years of 2010 and 2011, which could be explained by a true higher incidence, by improved diagnostic methods, and increased efforts in detecting and reporting HFMD. During the H1N1 pandemic in 2009, the transmission of respiratory viruses among children in Guangzhou was greatly reduced by massive use of face masks, school closures and reduction of outdoor activities. Such preventive measures and media attention could partly explain the lower HFMD incidence in 2009.

We observed a positive linear relationship between temperature and HFMD incidence as well as between relative humidity and HFMD incidence, which is consistent with the finding of other recent studies in Fukuoka, Japan [6] and Hong Kong [14]. Our results also indicate that the association of HFMD incidence to temperature features a rapid increase below and flattening above 25°C. This is similar to the previous finding from Tokyo, Japan [15], which reported a negative association between the number of days per week of average temperature above 25°C and HFMD incidence. In contrast to our present findings, the Japanese study did not find rainfall as a significant risk factor for HFMD incidence [6]. This discrepancy might be partially due to the fact that relative humidity and rainfall were adjusted for simultaneously in our model to avoid potential collinearity. We also fitted a model including both relative humidity and rainfall, but rainfall showed no significant association with HFMD incidence. In addition, we found no statistically significant association between wind speed and HFMD which is not consistent with the Hong Kong study [14]. A report from Hong Kong Observatory showed that the average wind speed of Hong Kong for the period 1981–2010 was 3.1 m/s [32] which was greater than the average wind speed of Guangzhou during the study period (Table 1). It is possible that there may be a threshold effect of wind speed on HFMD, which the wind speed of Guangzhou did not exceed.

Generally, the association between the incidence of HFMD and local meteorological variables corresponds to the biological plausibility, i.e., what is known about the factors influencing the survival of enteroviruses. Previous studies carried out in laboratories show that the effect of temperature on enteroviruses depends on whether the circulating strains are temperature sensitive or resistant [33,34]. Likewise, the survival of enteroviruses, such as poliovirus and human rotavirus, was enhanced at higher levels of relative humidity [35,36]. The same biological effects may apply to the HFMD-related enteroviruses, but has not been reported yet. Furthermore, the geographical distribution of EV71 strains in surface water was similar to that of clinical stool samples [37,38], suggesting that water serves as a potential reservoir and vehicle of the viruses, and thus high humidity and rainfall play an important role in HFMD infection. On the other hand, several studies indicate that weather conditions are associated with changes in human contact behaviors, which could affect the incidence of HFMD [39,40]. For example, temperature and relative humidity are higher in summer when more people are likely to spend their time out of the house in more crowded or air-conditioned environments. This may lead to increases in contact frequency among children.

Our analyses suggest that the effects of temperature and humidity did not vary significantly in different age groups. This is contradicting the finding that children aged <1 year have higher levels of antibodies [20,41]. A study in Taiwan suggested that attending a child daycare center or kindergarten (in our study: 3–5 years old) significantly increased the likelihood of EV71 infection [42], likely due to increased crowdedness [1,42]. However, this finding in Taiwan is not consistent with our study. A possible explanation is that the Chinese Ministry of Health and local governments have initiated several measures to prevent and control the epidemic of HFMD in institutional settings since 2008, such as disinfection of toys, use of sanitary products and tableware, morning checks, hand-washing interventions, a case isolation system and school closures [43]. Consequently, the fact that the proportion of institutional HFMD cases decreased markedly over years could be related to the implementation of these preventive measures, although the incidence of HFMD kept increasing over the past four years. Demographic changes in Guangzhou city such as population growth, urbanization, the increasing reliance of working parents on community nurseries, may also affected HFMD incidence in younger children.

We found that HFMD activities are better explained when meteorological variables are used with one week lag, which is consistent with previous findings in Hong Kong. This finding is not surprising as one week lag probably matches the incubation period of enteroviruses and the potential delay in parental awareness of and response to clinical symptoms of children.

There is scientific consensus that climate change will negatively influence human health in multiple ways. An emerging approach addresses a broader spectrum of health risks due to the social, demographic, and economic disruptions caused by climate changes [44-48]. Global warming may prolong the primary peak period of HFMD in summer, and magnify the second peak in autumn or early winter [5,49,50]. Furthermore, global climate change will increase the frequency and severity of extreme weather events, such as rainstorms, floods and droughts. In addition, there may be potential climate-forced crowding in temporary shelter that could accelerate the spread of HFMD. Overall, we expect the frequency and intensity of HFMD outbreaks are expected to increase in the foreseeable future as a result of climate change.

One limitation of this study is that we used weekly meteorological data rather than daily data, because weekly measures were the minimum unit of measurement released by the National Meteorological Information Center. This may affect the accuracy of exposure assessment, and a further investigation using daily data is needed in future. Another limitation is that we were unable to differentiate between clinically diagnosed and laboratory confirmed cases reported to China CDC. Therefore, it was not possible to do a sensitivity analysis restricted to laboratory-confirmed cases.

## Conclusions

This study provides quantitative evidence that the incidence of HFMD cases in children was associated with higher average temperature and relative humidity. The delayed effects of temperature and humidity on HFMD are consistent with the incubation period of enteroviruses and the potential delay in parental awareness of and responses to the sickness of children. This information could be helpful in predicting the scale of outbreaks, guiding health resource allocation and building public health preparedness and intervention strategies. Similar studies in other geographical areas and over longer time periods are needed to better understand the impacts of meteorological variables on HFMD.

## Abbreviations

CDC: Center for disease control and prevention; CI: Confidence interval; EV71: Human enterovirus 71; GAM: Generalized additive model; HFMD: Hand, foot, and mouth disease; PACF: Partial autocorrelation function.

## Competing interests

The authors declare that they have no competing interest.

## Authors’ contributions

YH, TD participated in the design, performed data analysis and interpretation, and drafted the manuscript. SY, JG, GX, CH participated in the design and data interpretation of results and helped to finalize the manuscript. YH participated in the design and supervised the study, participated in interpretation of results, and helped to finalize the manuscript. All authors have read and approved the contents of the final version.

## Authors’ information

Co-First authors: Yong Huang and Te Deng.

## Pre-publication history

The pre-publication history for this paper can be accessed here:

http://www.biomedcentral.com/1471-2334/13/134/prepub
